# A Comparative Study of SMN Protein and mRNA in Blood and Fibroblasts in Patients with Spinal Muscular Atrophy and Healthy Controls

**DOI:** 10.1371/journal.pone.0167087

**Published:** 2016-11-28

**Authors:** Renske I. Wadman, Marloes Stam, Marc D. Jansen, Yana van der Weegen, Camiel A. Wijngaarde, Oliver Harschnitz, Peter Sodaar, Kees P. J. Braun, Dennis Dooijes, Henny H. Lemmink, Leonard H. van den Berg, W. Ludo van der Pol

**Affiliations:** 1 Brain Centre Rudolf Magnus, Department of Neurology and Neurosurgery, University Medical Centre Utrecht, Utrecht, The Netherlands; 2 Brain Centre Rudolf Magnus, Department of Neurology and Child Neurology, University Medical Centre Utrecht, Utrecht, The Netherlands; 3 Department of Genetics, University Medical Centre Utrecht, Utrecht, The Netherlands; 4 Department of Genetics, University Medical Centre Groningen, Groningen, The Netherlands; University of Edinburgh, UNITED KINGDOM

## Abstract

**Background:**

Clinical trials to test safety and efficacy of drugs for patients with spinal muscular atrophy (SMA) are currently underway. Biomarkers that document treatment-induced effects are needed because disease progression in childhood forms of SMA is slow and clinical outcome measures may lack sensitivity to detect meaningful changes in motor function in the period of 1–2 years of follow-up during randomized clinical trials.

**Objective:**

To determine and compare SMN protein and mRNA levels in two cell types (i.e. PBMCs and skin-derived fibroblasts) from patients with SMA types 1–4 and healthy controls in relation to clinical characteristics and *SMN2* copy numbers.

**Materials and methods:**

We determined SMN1, SMN2-full length (SMN2-FL), SMN2-delta7 (SMN2-Δ7), GAPDH and 18S mRNA levels and SMN protein levels in blood and fibroblasts from a total of 150 patients with SMA and 293 healthy controls using qPCR and ELISA. We analyzed the association with clinical characteristics including disease severity and duration, and *SMN2* copy number.

**Results:**

SMN protein levels in PBMCs and fibroblasts were higher in controls than in patients with SMA (p<0.01). Stratification for SMA type did not show differences in SMN protein (p>0.1) or mRNA levels (p>0.05) in either cell type. *SMN2* copy number was associated with SMN protein levels in fibroblasts (p = 0.01), but not in PBMCs (p = 0.06). Protein levels in PBMCs declined with age in patients (p<0.01) and controls (p<0.01)(power 1-beta = 0.7). Ratios of SMN2-Δ7/SMN2-FL showed a broad range, primarily explained by the variation in SMN2-Δ7 levels, even in patients with a comparable *SMN2* copy number. Levels of SMN2 mRNA did not correlate with *SMN2* copy number, SMA type or age in blood (p = 0.7) or fibroblasts (p = 0.09). Paired analysis between blood and fibroblasts did not show a correlation between the two different tissues with respect to the SMN protein or mRNA levels.

**Conclusions:**

SMN protein levels differ considerably between tissues and activity is age dependent in patients and controls. SMN protein levels in fibroblasts correlate with *SMN2* copy number and have potential as a biomarker for disease severity.

## Introduction

Hereditary proximal spinal muscular atrophy (SMA) is caused by survival motor neuron (SMN) protein deficiency due to homozygous deletion of the *SMN1* gene [1]. A second semi-homologous *SMN* gene (*SMN2*) contains a crucial single nucleotide substitution that alters mRNA splicing, resulting in the absence of exon 7 in the large majority of SMN2 mRNA transcripts [1, 2]. Copy number variation of *SMN2* is the most important modifier of disease severity [3].

SMN protein is ubiquitously expressed and has generic functions as part of a number of protein complexes in addition to tissue-specific functions, including mRNA processing and splicing [4–6], axonal transport [7, 8] and ubiquitination homeostasis [9, 10]. Quantification of SMN protein and mRNA levels may be useful as a biomarker for SMA severity and to monitor the response to experimental strategies designed to increase SMN protein [11–14] and changes in SMN expression have already been used to study the potential of SMN-inducing drugs as a treatment for SMA [11, 14–19].

Various methods have been developed to (semi-) quantify SMN protein and mRNA levels. Southern and western blotting [20–26], imaging-flow cytometry [27, 28] and simple-cell-immuno-assays [29, 30] were used in studies to investigate SMN levels in lymphoblasts, peripheral blood mononuclear cells (PBMCs) and fibroblasts in small cohorts of SMA patients. qPCR [22, 31, 32] and ELISA [12, 15, 18, 22, 33–35] have shown their applicability in larger studies with patients participating in randomized controlled trials with SMN inducing therapies such as valproic acid and salbutamol [11, 15, 19]. Recently, electrochemiluminescence-based immunoassay (ECLIA or ECL) was introduced for measurements of SMN levels in small amounts of whole blood [32, 36, 37].

Reduced SMN levels have been found in a large variety of tissues in SMA mouse models, including muscle [33, 38], myotubes [39], brain [33, 38, 40], astrocytes [41], spinal cord [33, 36, 38, 40], Schwann cells [42], skin [33] and liver [33]. In humans, similar findings have been reported in a smaller number of tissues that include brain [43], muscle [43], whole blood [32, 36], PBMCs [12, 15, 18, 22, 29, 33, 34], fibroblasts [20, 26, 29] and buccal cells [36, 37]. SMN protein levels have also been investigated in body fluids, most notably in cerebrospinal fluid as an exploratory biomarker in a phase 1 study of intrathecal administration of antisense oligonucleotides [44], but also in urine, plasma and saliva [33, 36, 37]. However, the extent to which tissues differ in SMN mRNA and protein concentrations in humans is still largely unknown [45].

A second unaddressed issue is how aging affects SMN levels. Possible age-dependent changes in levels of SMN have been reported in SMA mice [33]. Previous patient studies have included far more children than adults with SMA and this limitation in age range has precluded a definite conclusion regarding the effect of age on SMN levels [11, 12, 14–18, 22]. We therefore determined SMN protein and mRNA levels in blood and skin-derived fibroblasts from a large cohort of children and adults with SMA and matched healthy controls using ELISA and qPCR methodology.

## Materials and Methods

### Study population

We performed a cross-sectional, single visit, single-center, nationwide study on SMA in The Netherlands. Inclusion criteria were a genetically confirmed diagnosis of SMA according to the diagnostic criteria defined by the SMA Consortium, i.e. a homozygous deletion of the *SMN1* gene, or a hemizygous deletion with an additional pathogenic point mutation in the second *SMN1* allele [1, 46, 47]. We used age at onset and acquired motor milestones to define SMA types 1–4 as described previously [46, 48, 49]. Patients with SMA type 1 had an onset of muscle weakness before the age of 6 months and were never able to sit independently. Patients with SMA type 2 had an onset between the age of 6 and 18 months and learned to sit but not to walk independently. Patients with SMA type 3 had an onset after the age of 18 months, learned to walk independently at some stage in life. Onset in patients with SMA type 4 occurred after the age of 30. In case of discrepancy between age at onset and reached motor milestones, the latter determined the final diagnosis. We included 6 adult patients with onset before 6 months of age and who survived infancy but never learned to sit independently. This unusual SMA type 1 phenotype (‘type 1c’) has been reported before [50–53]. Disease duration was calculated as time between the age of first symptoms and date of enrolment. The healthy control group consisted of 293 children and adults without neurological disease or a current infection.

We used Medical Research Council (MRC) sum scores of 38 individual muscle groups to document muscle strength. Each muscle was given a score ranging from 1 to 5 (MRC sum score range 38–190). The Hammersmith Functional Motor Scale Expanded (HFMSE) was used to document motor function [54].

The Medical Ethical Committee of the University Medical Center Utrecht approved the study protocol (protocol number 09–307) and all participants and/or legal representatives gave written informed consent.

### *SMN* copy number analysis

We determined the total number of *SMN1* and *SMN2* gene copies in patients by Multiplex Ligation-dependent Probe Amplification (MLPA) analysis using SALSA MLPA kits P021-A2 and P060-B2, according to the manufacturer’s protocol (www.mrcholland.com).

### PBMCs

Peripheral blood mononuclear cells (PBMCs) were isolated from 5–10 ml Lithium-Heparin-blood samples using Lymfoprep (Axis Shield PoC AS, Oslo, Norway) and complete Lysis-M EDTA-free buffer (Roche Diagnostics GmbH, Mannheim Germany). Isolation was performed within 4 hours of sample collection. PBMC counts from samples ranged from 1.26x10^7^ to 3.3x10^7^ cells.

We tested three protein extraction buffers using PBMCs that were pelleted and re-suspended in 100 μL of each buffer. Comparison between Complete Lysis-M EDTA-free buffer, RIPA buffer and ENZO lysis buffer showed comparable inter-well variability (Complete Lysis-M EDTA-free buffer: mean 6.0% CV; RIPA buffer: 5.5% CV; ENZO lysis buffer mean 5.9% CV). Mean inter-plate variability was 35% (range 8.7–90%). We used Complete Lysis-M EDTA-free buffer (Roche Diagnostics GmbH, Mannheim Germany) for all samples. Lysates were stored at -80°C in aliquots of 100–200 μl.

### Fibroblasts

Patient-derived fibroblasts were generated from explants of 3 mm dermal biopsies. After 1–2 weeks, fibroblast outgrowths from the explants were passaged with trypsin and frozen. Fibroblasts were cultured in standard fibroblast medium (Dulbecco’s modified eagle medium containing 10% fetal bovine serum and 0.5% penicillin and streptomycin), and lysed with Complete Lysis-M EDTA-free buffer (Roche Diagnostics GmbH, Mannheim Germany). Lysates were stored at -80°C.

### Measurements of SMN protein concentrations

We determined total soluble protein concentrations of the samples in triplo using protein assay with Bicinchoninic Acid (#23227, Pierce BCA Protein Assay Kit; Thermo Scientific, Rockford, IL) and generated standard curves using dilutions (0.1–3.0 mg/ml) of bovine serum albumin (BSA) (A7906-500G, Sigma Alderich Chemie, Steinheim, Germany).

We normalized samples to 1 gram total soluble protein from BCA-analysis. SMN protein levels in PBMCs and fibroblasts were quantified using the standardized SMN ELISA (2012, #ADI-900-209, Enzo Life Sciences, Farmingdale, NY) [33, 34] and expressed as nanogram per 1 gram of total protein.

### Quantitative polymerase chain reaction of SMN transcripts

We used PAXgene blood RNA tubes (BD Biosciences, San Jose, CA, USA) for storage and stabilization of RNA from peripheral blood. RNeasy Mini Kit (Qiagen, Dusseldorf, Germany) was used to extract RNA from blood and fibroblasts.

RNA concentration was determined by absorbance determination and quality was assessed by nanodrop analysis (absorbance of 230, 260 and 280nm). A ratio (260/280) of ±2.0 was accepted as pure. Quality and integrity control of PAXgene samples was performed with an Agilent 2100 bioanalyzer and 90% of samples met the quality criterion of RNA Integrity Number >7 (mean 8.1, median 8.2, range 4.4–9.2). We used Taqman Gold RT-PCR kit (Applied Biosystems, No.N808-0232) for the reverse transcription of 500 ng RNA to cDNA.

We used 2 control primer sets (glyceraldehyde 3-phosphate dehydrogenase (GAPDH) and 18S), and three SMN-primer sets on each sample. External standard constructs and primers for SMN1-FL, SMN2-FL, SMN2-Δ7, GAPDH and 18S were designed as reported previously [31]. *GAPDH* and *18S* genes were both used for analysis (median intra-sample variation 0.8 and 1.1% respectively (range 0.1–3.3)) by means of the geometric mean of the two genes [55]. Standard curves were determined with Avogadro’s number. The real-time Taqman PCR reactions were carried out in 1x Taqman universal PCR mastermix (Applied Biosystems, P/N 4326708), 1x Primer-Probe mix (Applied Biosystems), with an input of 10 ng cDNA. qPCR was carried out as described previously [31]. Analysis was performed on Sequence Detection System v2.3 (Applied Biosystems). All samples were normalized against 10^5^ molecules of the reference genes. Outliers in all expression sets per patient were excluded when they failed the Grubb’s test or deviated by >1SD from the sample mean.

Calculated ratios between transcripts of SMN2-FL and SMN2-Δ7 (SMN2-Δ7/SMN2-FL) were used to analyze the dose-effect of the *SMN2* gene copy number variation.

### Sample size and statistics

A sample size of 324 (allocation 1:2) was needed to reach 90% power to detect a difference in means between SMA patients and controls in SMN protein levels in PBMCs, using a two-group independent t-test with a 0.05 two-sided significance level based upon results from Crawford et al[22]. Post-hoc power analysis of 135 PBMC samples and their correlation with *SMN2* copy number, age and SMA type showed a power of 80% using a two-sided ANOVA (alpha 0.01; partial etha^2^ 0.15). Post-hoc power analysis of 87 fibroblast samples and their correlation with *SMN2* copy number, age and SMA type showed a power of 87% using a two-sided ANOVA (alpha 0.01; partial etha^2^ 0.43).

Normality was tested with Kolmorogov-Smirnov and Shapiro-Wilk tests. Mean, medians and SD for continuous variables and proportions for categorical variables were calculated. Correlation matrixes were analyzed using the Spearman’s rho. Univariate and multivariate tests including dichotomous data were performed using logistic regression. Multivariate analyses were checked and corrected for co-linearity. Comparison of data between SMA types and between patients and controls was performed using Kruskal-Wallis (KW) test or Chi-square analysis. Multivariate analysis was performed with linear regression including bootstrapping analysis. P-values ≤0.05 were considered significant.

We used SPSS (IBM SPSS Statistics version 19, Inc., Chicago, IL) for statistical analysis.

## Results

### Clinical characteristics

We included 150 patients with SMA type 1–4 and 293 healthy controls. Clinical characteristics are summarized in Tables 1 and 2. *SMN2* copy numbers correlated with SMA type (Chi^2^ p<0.001). Age and disease duration differed between SMA types, and *SMN2* copy numbers (KW p<0.01). Three patients used a stable dose of valproate at the time of this study. One patient had discontinued use of valproate more than one year before inclusion. None of the other patients were on other potentially SMN-inducing therapies (e.g. salbutamol).

**Table 1:** Baseline characteristics of patients in PBMC study.

	Type 1^a^ (n = 18)	Type 2 (n = 60)	Type 3a^b^ (n = 26)	Type 3b (n = 26)	Type 4 (n = 5)	Controls (n = 229)
Gender (n) (F:M)	7:11	36:24	15:11	11:15	4:1	115:114
Mean age at inclusion in years (range)	10.6 (0.3–49.7)	19.6 (1–66.7)	36.8 (2.4–65.7)	38.8 (14–75)	51.2 (41–68.8)	32.7 (0.3–86)
Mean disease duration in years (range)	11.1 (0.1–48.4)	18.2 (0.3–64.8)	33.6 (1.2–62.2)	29.5 (2–71.4)	14.3 (7.5–24.2)	NA
Mean HFMSE (range)	0 (0–1)	8 (0–35)	17 (0–44)	36 (4–66)	48 (43–53)	ND
Mean MRC sum score (range)	51 (34–62)	89 (43–140)	104 (56–160)	146 (100–167)	147 (121–162)	ND
*SMN2* copy number (n)						
*2*	4	3	0	0	0	ND
*3*	13	52	14	3	0	ND
*4*	1	5	10	21	4	ND
*5*	0	0	0	2	0	ND

PBMC = Peripheral blood mononuclear cell; F = female; M = male; SMN = survival motor neuron; HFMSE: Hammersmith Functional Motor Scale Expanded; MRC = Medical Research Council; ND = not determined; NA = not applicable

^a^ = Six patients with SMA type 1 had survived infancy at time of inclusion

^b^ = One patient had a heterozygous *SMN1*-deletion and a pathogenic point mutation resulting in stop codon in exon 4

**Table 2:** Baseline characteristics of patients in fibroblast study.

	Type 1^a^ (n = 5)	Type 2 (n = 19)	Type 3a^b^ (n = 10)	Type 3b/4 (n = 6)	Controls (n = 47)
Gender (n) (F:M)	3:2	11:8	7:3	1:5	26:21
Mean age at inclusion in years (range)	15.3 (0.4–42.2)	20.1 (1–66.7)	34.6 (6–61.9)	39.1 (14–54.7)	56.1 (25–77)
Mean disease duration in years (range)	17.5 (0.3–41.2)	19.8 (2.6–64.8)	28.9 (4.4–60)	26.1 (2–39.4)	NA
Mean HFMSE (range)	0 (0)	8 (0–23)	19 (0–45)	43 (14–64)	ND
Mean MRC sum score (range)	37 (34–40)	94 (52–121)	123 (59–160)	151 (141–163)	ND
*SMN2* copy number (n)					
*2*	2	0	0	0	ND
*3*	4	17	3	0	ND
*4*	0	2	5	5	ND
*5*	0	0	0	1	ND

F = female; M = male; SMN = survival motor neuron; HFMSE: Hammersmith Functional Motor Scale Expanded; MRC = Medical Research Council; ND = not determined; NA = not applicable

^a^ = Three patients with SMA type 1 had survived infancy at time of inclusion

^b^ = One patient had a heterozygous *SMN1*-deletion and a pathogenic point mutation resulting in stop codon in exon 4

### Sample reproducibility

SMN protein levels in PBMCs and fibroblasts showed sample variability, similar to previous reports [22, 33]. Measurements of total protein used for normalization showed an inter-well variation of 4.2% (range 0–19%) and inter-plate variation of 3.2% (range 0.2–8.7%) with a mean day-by-day variation of 7.3% (range 0.7–21.5%). Analyses of inter-well coefficients of variance (CV) ranged from 0.2–26% (mean 5.3%) for SMN protein normalized for total protein levels. Mean inter-plate variability was 10% (range 0.5–100%; median 6.2%). After one extra freeze-thaw cycle, CV ranged from 1–60% within protein samples and CV between plates increased to 40%. Analyses were therefore only performed once after storage, without any extra freeze-thaw episodes to prevent protein changes due to freeze-thaw effects. Overall time in storage at -80°C varied per protein sample (median = 4 months; range 0–33 months).

CV of mRNA expression levels was good (<5%). Mean inter-well variability in expression levels of SMN1, SMN2-FL, and SMN2-Δ7 was 1.1%, 1.2%, and 0.8% respectively in blood and 1.2%, 0.7%, and 0.6% in fibroblast samples. Mean inter-plate variability was 2.1%, 3.0%, and 2.5% respectively for SMN1, SMN2-FL, and SMN2-Δ7 in both cell types (range 1.6–4.4%).

### SMN protein analysis

**Fig 1:**
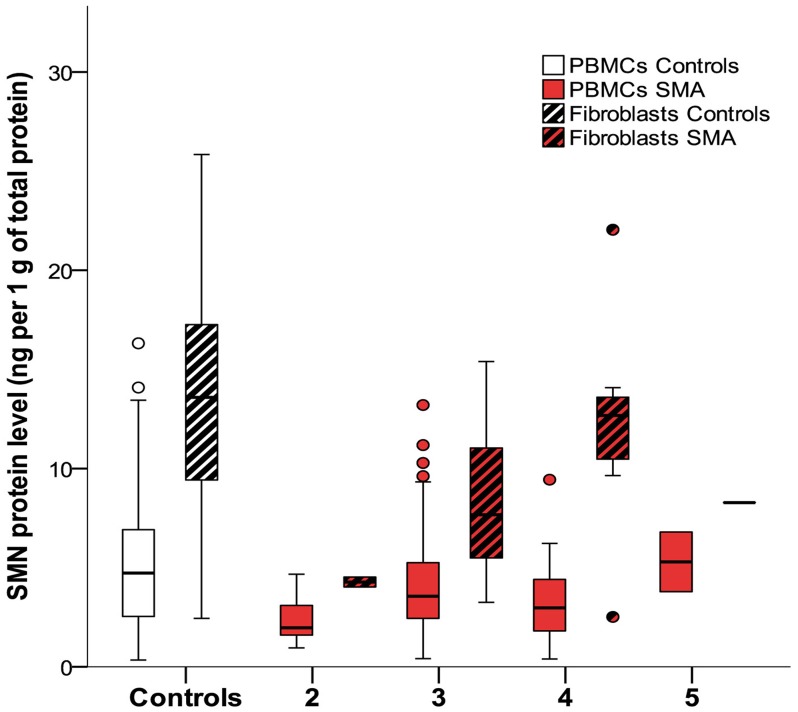
SMN protein levels in PBMCs and fibroblasts from patients and controls and effect of *SMN2* copy numbers. Mean SMN protein levels are higher in controls compared to patients. SMN protein levels in PBMCs did not differ significantly between patients with 2, 3, 4 or 5 *SMN2* copies (p = 0.06). Higher *SMN2* copy number is associated with higher levels of SMN protein in fibroblasts (p = 0.01). Boxplot elements represent: median (line in the middle), 1^st^ en 3^rd^ quartile (bottom and top of the box), highest case with 1.5 time inter-quartile range (bottom and top whisker) and outliers (dots).

Mean SMN protein levels were higher in controls compared to SMA patients in PBMCs and in fibroblasts (both log regression p<0.01) (Fig 1 and Table 3). There was a trend towards differences in SMN concentrations in PBMCs after stratification for *SMN2* copy number (KW p = 0.06). Higher *SMN2* copy number was associated with higher levels of SMN protein in fibroblasts (KW p = 0.01) (Fig 1). SMN protein levels did not differ between SMA types (PBMCs KW p = 0.18; fibroblasts KW p = 0.34).

**Table 3:** Levels of SMN protein in PBMCs and fibroblasts.

	*PBMCs*		*Fibroblasts*	
	*SMA (n = 135)*	*Controls (n = 229)*	*SMA (n = 40)*	*Controls (n = 47)*
SMN protein levels* **Mean *±SD (range)***	3.7 ± *2*.*4 (0*.*4–13*.*2)*	5.3 ± *3*.*6 (0*.*3–18*.*3)*	8.8 ± *4*.*3 (2*.*5–22*.*1)*	13.4 ± *5*.*6 (2*.*4–25*.*8)*

PBMCs = Peripheral blood mononuclear cells; ND = not determined

* = nanogram per 1 gram total protein

SMA severity reflected by HFMSE score and MRC sum scores did not correlate with SMN protein levels in PBMCs (Spearman’s rho p = 0.15 and p = 0.6 respectively), but did correlate with SMN protein levels in fibroblasts (Spearman’s rho p = 0.004 and p = 0.04).

**Fig 2:**
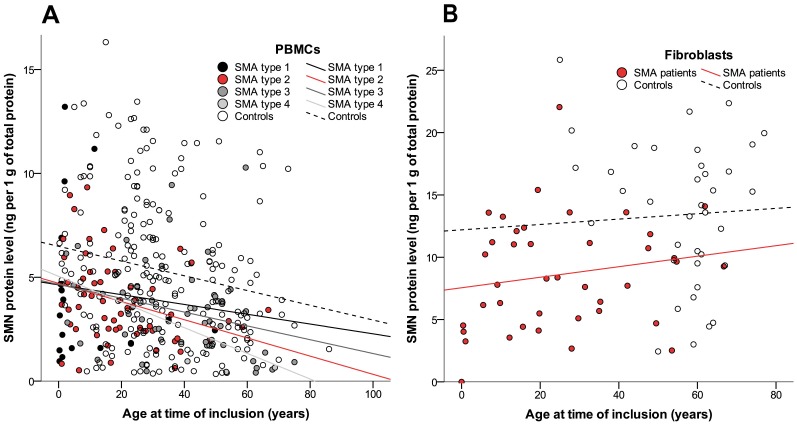
SMN protein levels in relation to age. (A) Levels of SMN protein in PBMCs decline with age (p<0.01) in patients and controls (Spearman rho correlation coefficient: patients -0.31; controls -0.21). (B) No correlation between age and SMN protein levels in fibroblasts in patients or controls (p = 0.43).

Disease duration and age at time of inclusion correlated inversely with SMN levels in PBMCs (both Spearman’s rho -0.31, p<0.01) (Fig 2A). This correlation between age as well as disease duration, and SMN levels was present in patients and controls (both p<0.01) and persisted when SMA types 2 or 3 were analyzed separately (type 1 Spearman’s rho 0.2, p = 0.4; type 2 Spearman’s rho -0.3, p<0.05; type 3 Spearman’s rho -0.4 p<0.01) (Fig 2A). There was no correlation of SMN levels and age at time of inclusion in fibroblasts (p = 0.43) (Fig 2B).

Paired analysis of SMN levels was possible using PBMCs and fibroblasts from 33 patients with SMA. SMN protein concentrations were higher in fibroblasts than PBMCs (log regression p<0.01). Protein levels in PBMCs and fibroblasts did not correlate (Spearman’s rho p = 0.7).

### mRNA expression analysis

Two blood samples from patients with SMA were excluded from analysis due to low quality of RNA (RIN<4), and 8 were excluded because of undetectable mRNA levels of GAPDH and/or 18S.

**Fig 3:**
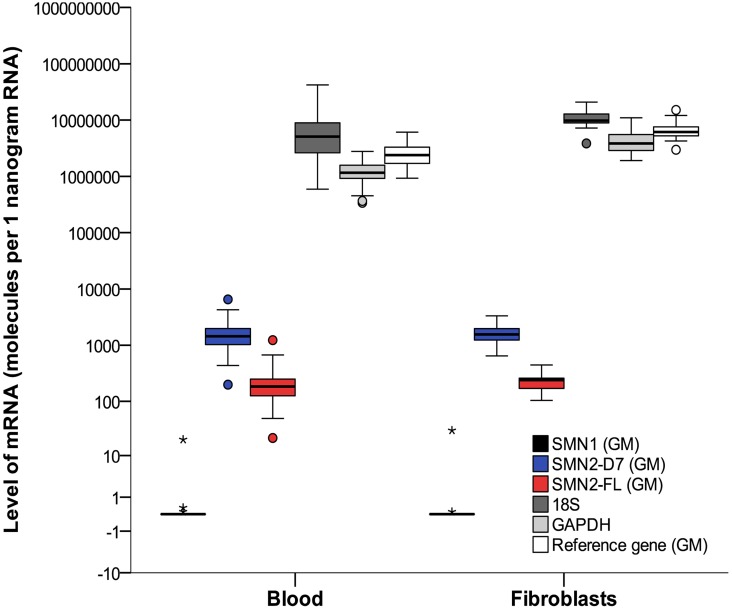
mRNA levels of SMN and reference genes. Analysis of mRNA was performed in blood and fibroblasts from patients with SMA. Boxplots represent mRNA levels of SMN1, SMN2-FL and SMN2-Δ7 normalized by the geometric mean of the two reference genes (GAPDH and 18S). The reference gene plot (white bar) represents the geometric mean (GM) of GAPDH (light grey bar) and 18S (dark grey bar). For reasons of clarity, individual levels of GAPDH and 18S are presented as well. Levels of SMN2-FL and SMN2-Δ7 did not differ between blood and fibroblasts (p>0.05). One patient had a heterozygote deletion and an additional point mutation of the *SMN1* gene, represented by a SMN1-mRNA level of 20 molecules per 1 nanogram of RNA shown by the asterisk (SMN1-levels in 6 controls ranging from 150–350 molecules per 1 nanogram (data not included in this report)). Boxplot elements represent: median (line in the middle), 1^st^ en 3^rd^ quartile (bottom and top of the box), highest case with 1.5 time inter-quartile range (bottom and top whisker) and outliers (dots and asterisks). SMN2-D7 = SMN2-Δ7.

SMN1 mRNA could be detected at low levels in blood and fibroblasts from the one patient with a heterozygous deletion of *SMN1* and an additional point mutation in the second allele, but was absent in all other patients (Fig 3).

**Fig 4:**
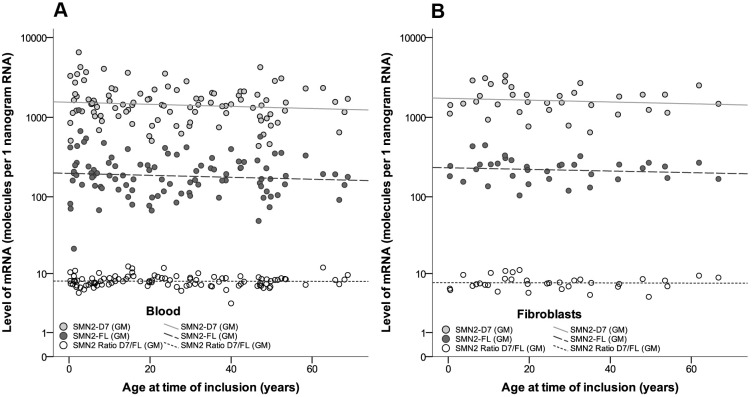
SMN mRNA transcript levels in blood and fibroblasts from patients with SMA in relation to age. (A) SMN2 mRNA expression levels in blood from patients with SMA. SMN2-Δ7 levels were significantly higher than SMN2-FL levels. SMN2-FL and SMN2-Δ7 levels in blood did not correlate with age (p = 0.35). (B) SMN2 mRNA expression levels in fibroblasts in patients with SMA. SMN2-Δ7 levels were significantly higher than SMN2-FL levels. Data shown are normalized to geometric mean (= GM) of GAPDH- and 18S-reference genes. SMN2-D7 = SMN2-Δ7.

**Fig 5:**
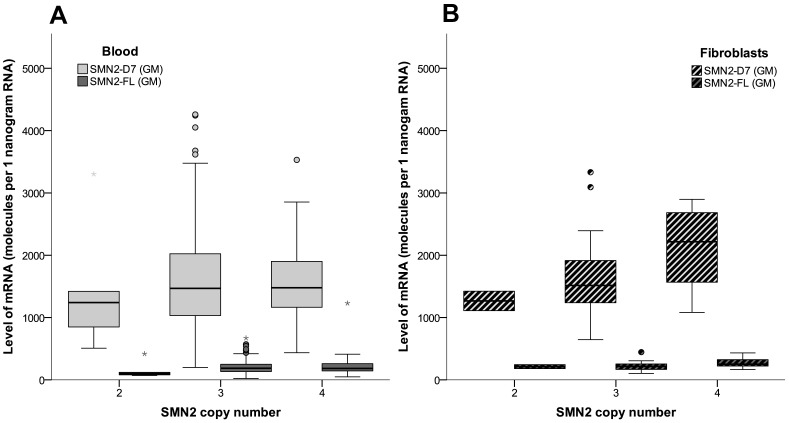
SMN2 mRNA expression levels in blood and fibroblasts from patients with SMA in relation to SMN2 copy number. Levels of SMN2-FL and SMN2-Δ7 in relation to *SMN2* copy number in blood (Panel A; KW SMN2-FL p = 0.7; KW SMN2-Δ7 p = 0.3) and fibroblasts (Panel B; KW SMN2-FL p = 0.3; KW SMN2-Δ7 p = 0.09) from patients with SMA. Data shown are normalized to the geometric mean (= GM) of GAPDH- and 18S-reference genes. Boxplot elements represent: median (line in the middle), 1^st^ en 3^rd^ quartile (bottom and top of the box), highest case with 1.5 time inter-quartile range (bottom and top whisker) and outliers (dots and asterisks). SMN2-D7 = SMN2-Δ7.

Expression levels of SMN2-FL and SMN2-Δ7 in blood correlated with each other (Spearman’s rho 0.95, p<0.001). There was no effect of gender on expression of SMN2-FL or SMN2-Δ7 (p = 0.3). Levels of SMN2-FL and SMN2-Δ7 did not show a correlation with age at time of inclusion (Fig 4A), SMA type or *SMN2* copy number (Fig 5A) in blood (age p = 0.35; SMA type KW p = 0.7; *SMN2* copy number KW p = 0.3). Ratios of SMN2-Δ7/SMN2-FL ranged from 4.6 up to 12.5, mostly explained by variation in SMN2-Δ7 transcript levels. Disease severity, reflected by clinical scores (MRC sum score and HFMSE), did not correlate with mRNA expression levels of SMN2-FL or SMN2-Δ7 (p = 0.5 and p = 0.7, respectively).

Levels of SMN2-FL and SMN2-Δ7 could be analyzed in fibroblasts from 35 subjects with SMA (Table 4). Levels of SMN2-FL correlated with levels of SMN2-Δ7 (Spearman’s rho 0.74, p<0.001). Ratios of SMN2-Δ7/SMN2-FL ranged from 4.6 to 11. We did not find associations between any of the transcript levels and age (p>0.2; Fig 4B), disease duration (p = 0.4), SMA type (KW p = 0.2), or disease severity reflected by current HFMSE and MRC sum score (p = 0.8 and p = 0.3). Levels of SMN2-FL and SMN2-Δ7 were higher in patients with 4 *SMN2* copies compared to 2 or 3 copies, but this was not significant (log regression p = 0.09) (Fig 5B).

**Table 4:** Levels of SMN mRNA in blood and fibroblasts from patients with SMA.

	*Blood*	*Fibroblasts*
SMN1* ***Mean ±SD (range)***	0.4 *± 4*.*3 (0–20)*	1.1 *± 6*.*5 (0–29)*
SMN2-Δ7* ***Mean ±SD (range)***	1666 *± 1000 (198–6525)*	1745 *± 688 (646–3332)*
SMN2-FL* ***Mean ±SD (range)***	219 *± 158 (22–1230)*	231 *± 78 (104–1445)*

ND = not determined

* = Levels presented as molecules per 1 nanogram RNA referenced against the geometric mean of 18S and GAPDH

Paired samples of transcript levels of *SMN2* genes and reference genes (*GAPDH* and *18S*) in both blood and fibroblasts were available from 23 patients. Expression levels of SMN2-FL were higher compared to SMN2-Δ7, in blood as well as in fibroblasts (Fig 4A and 4B). Mean levels of SMN2-FL and SMN2-Δ7 did not differ between blood and fibroblasts (log regression p = 0.7; independent t-test p = 0.6) (Table 4). There was no correlation between blood or fibroblast expression levels for the separate transcripts (Spearman’s rho = -0,2; p = 0.50). Correction for age or stratification for *SMN2* copy number did not alter results.

Paired analysis of protein and transcript levels in blood was possible in 99 subjects, with 35 samples available for fibroblast analysis. There was no correlation between SMN protein and SMN mRNA expression levels in blood (Spearman’s rho 0.10, p = 0.3 (corrected for age)), nor in fibroblasts (Spearman’s rho 0.10, p = 0.6 (corrected for age)).

## Discussion

This is the first comparative study of SMN protein and mRNA levels in PBMCs and fibroblasts in a large cohort of patients with SMA. In addition to the reduced levels of SMN protein and mRNA in patients with SMA, we found an association of *SMN2* copy number with SMN protein in fibroblasts only, although we observed a similar trend in PBMCs. There was an age- and disease duration-dependent decline of SMN protein concentrations in PBMCs. Finally, we did not find a correlation of SMN mRNA or protein between blood and fibroblasts, suggesting important expression differences between tissues or cell types.

SMN protein and mRNA levels are obvious biomarker candidates both for disease severity and for efficacy of experimental treatment strategies in SMA. SMN levels have primarily been quantified in blood, first in PBMCs [12, 15, 18, 22, 29], and more recently in whole blood samples [32, 36]. We used the previously described and calibrated SMN-specific qPCR [22, 31, 32] and ELISA [12, 15, 18, 22, 33–35] techniques with minor modifications that previously (and also in our hands) showed good inter- and intra-sample variance. Both techniques offer the advantage of robust high throughput analysis of large numbers of samples in relatively small blood volumes. In contrast to previous studies that often used a single gene as reference (GAPDH [17, 22, 25, 26, 31, 32, 56–58], 18S [29], PKG1 [11, 16, 57], GUSB [17, 29, 57], PPIA [57], HRPLPO [11, 13, 16, 19], Beta-actin [26, 59], MLH1 [60], HPRT [60]), we used the geometric mean of two reference genes (GAPDH and 18S) to quantify SMN mRNA levels. Although this methodological modification complicates comparison between studies, results are less likely to be influenced by random variation in reference gene expression [55, 61]. Ideally, an even larger set of reference genes should be used for reference, but the relatively small blood volumes that can be obtained from the youngest children with SMA obviously complicates this.

Although SMN protein levels have been studied in many (experimental) cell types [12, 15, 18, 20, 22, 26, 29, 32–34, 36–43], there are no comparative studies of SMN expression in tissues that can be easily obtained. Significant differences in SMN protein levels have recently been found in platelets, red blood cells and PBMCs, which underlines the importance to investigate tissue-specific SMN expression [32, 36]. In this study we therefore determined and compared SMN expression in PBMCs and skin-biopsy derived fibroblasts. We found reduced levels of SMN mRNA and protein in both PBMCs and fibroblasts from patients with SMA compared to healthy controls. In line with previous observations [12, 18, 22, 29, 31, 33, 34] there was no association of SMN protein or mRNA levels in blood with SMA type [15, 18, 22, 29, 31–34], although there was a trend towards an association of SMN protein with *SMN2* copy number. Despite the significantly smaller sample size of fibroblasts compared to PBMCs, we found a correlation of SMN protein levels with *SMN2* copy number in fibroblasts, and also with clinical characteristics such as MRC sum and HFMSE scores. Our data therefore suggest that skin-derived fibroblasts may be a more robust cell type for SMN biomarker studies. The fibroblast study may have been underpowered to show a correlation with SMA type, since this is, although not perfectly, associated with *SMN2* copy number.

The lack of correlation of SMN levels between PBMCs and fibroblasts suggests important expression differences between tissues. This may be explained by differences in SMN concentrations required for normal development and function of specific cell types, and may for example be explained by variation in epigenetic modifications in stem cells or germ layers [62]. However, highly related cell types may have significantly different SMN protein levels, as shown by two recent studies using a new electrochemiluminescence (ECL) assay to detect SMN levels in whole blood [32, 36]. In these studies, platelets and red blood cells contributed most to SMN levels in whole blood (both cell types accounted for 40%), whereas SMN levels in PBMCs were relatively low (20% of total SMN) [32, 36].

Optimizing SMN quantification techniques is important for future clinical trials of SMN enhancing therapies, since findings in animal models for SMA suggest improved outcome upon increased peripheral SMN expression [43]. It has been suggested that the recently developed ECL has higher sensitivity to detect relevant differences in SMN expression, for example between patients with varying *SMN2* copy numbers or tissues, but this needs to be shown in comparative studies with an adequate sample size. ECL in whole blood may have the advantage of more straightforward sample processing that could reduce variation caused by PBMC processing methods[18, 33, 63, 64], storage conditions[34, 65] and extraction and lysis reagents [22, 33]. We rigorously applied predefined protocols to keep this variation limited. It was also recorded whether patients recently had a viral infection, since this may also cause variation in SMN levels [34, 65–67].

Our data show an age dependent decline of SMN protein levels in PBMCs in both patients with SMA and healthy controls. This confirms previous preliminary data from 2 studies including a total of 49 children and adults that suggested an effect of aging on SMN protein levels in PBMCs [34] and whole blood [32]. Meta- analysis of these studies with our results is not possible due to methodological differences, such as variation in laboratory techniques and patient characteristics, including SMA type, age-range, and the inclusion of data that reflect clinical severity. There are several explanations for this observed decline. Reduced SMN expression may be a feature of normal aging. Age-specific differences in SMN expression levels in humans have been reported previously. SMN expression is probably highest in the embryonic period and declines after birth [43, 68, 69]. It is not known whether a continuing decline with age could contribute to the slow deterioration of motor function that has been observed in adult patients[70, 71]. Another explanation may lie in changes in the relative PBMC composition during life [72]. We cannot exclude the possibility that a relative decline of specific mononuclear cells with high SMN expression in the course of life underlies our findings.

The strengths of our study are the size of both patient and control groups, the wide range of age and disease severity and the detailed clinical data, and the novel comparative approach. An apparent weakness of this study is the cross-sectional design that does not allow investigation of the individual rate of decline in SMN protein or expression levels. Future longitudinal studies should attempt to address changes in SMN expression in relation to age in individual patients and explore the added value of the ECL technique, ideally in a comparative study of whole blood, PBMCs and fibroblasts.

## Supporting Information

S1 STROBE Checklist (PDF)
